# Thyroid peroxidase antibodies and their role in predicting outcomes in Graves’ disease treatment

**DOI:** 10.3389/fendo.2025.1517283

**Published:** 2025-04-04

**Authors:** Klara Gewert, Geriolda Topi, Tereza Planck, Jan Calissendorff

**Affiliations:** ^1^ Department of Clinical Sciences, Lund University, Malmö, Sweden; ^2^ Department of Endocrinology, Skåne University Hospital, Malmö, Sweden; ^3^ Department of Endocrinology, Karolinska University Hospital, Stockholm, Sweden; ^4^ Department of Molecular Medicine and Surgery, Karolinska Institutet, Stockholm, Sweden

**Keywords:** Graves’ disease, hyperthyroidism (Graves’ disease), antithyroid drugs (ATD), relapse, remission, long-term follow-up, recurrence, radioiodine

## Abstract

**Introduction:**

Graves´ disease (GD) is the predominant cause of hyperthyroidism. Treatment options include antithyroid drugs (ATD), surgery, and radioactive iodine ablation (RI). Although thyroid peroxidase antibodies (anti-TPO) are prevalent in patients with GD, their role in driving relapse or hypothyroidism after treatment in patients with GD remains unclear. This study aimed to determine if patients with anti-TPO at GD diagnosis are more likely to relapse after ATD or RI treatment, and if patients with anti-TPO are at increased risk of developing hypothyroidism post-ATD treatment.

**Methods:**

This was an observational, non-interventional retrospective registry study, which included 712 patients treated for GD at a single center in Sweden during 2002-2018.

**Results:**

After therapy with ATD, there was no difference in relapse rate between patients with (37.0%) or without (38.4%) anti-TPO at GD diagnosis. Age <40 years was a risk factor for relapse after ATD (p<0.0001). Presence of anti-TPO at diagnosis was associated with reduced relapse rate after RI (13.9% vs. 24.6%; p=0.049). Development of hypothyroidism after discontinuation of ATD did not correlate with anti-TPO status at diagnosis (with anti-TPO: 17.3%; without anti-TPO: 20.8%). Increased risk of hypothyroidism was seen with ATD treatment for >2 years, p<0.05.

**Conclusion:**

Anti-TPO positivity at diagnosis of GD did not affect the relapse rate after ATD treatment but could be associated with a better long-term effect of RI. Anti-TPO did not increase the risk of hypothyroidism post-ATD therapy. Understanding risk factors of relapse or hypothyroidism can facilitate treatment choices and help physicians individualize management and follow-up strategies for patients with GD.

## 1 Introduction

Grave’s disease (GD) is the most common cause of hyperthyroidism (1). GD is caused by an interplay between endogenous, genetic, and environmental factors (2). It is an autoimmune disorder, in which thyroid-stimulating hormone receptor autoantibodies (TRAb) stimulate the thyroid stimulating hormone (TSH) receptor, leading to hyperthyroidism (2). Association with other autoimmune diseases has been reported (3, 4). The vast majority of patients with GD (90%-95%) are TRAb-positive (5). There are three types of TRAb; a stimulating autoantibody which causes GD, a blocking autoantibody, and a neutral one (6). However, measuring TRAb in routine clinical practice does not provide information on the biologic activity of these antibodies (7, 8).

Thyroid peroxidase antibodies (anti-TPO) are strongly associated with autoimmune hypothyroidism (5). Nevertheless, a significant proportion of patients with GD also test anti-TPO positive (2). Anti-TPO affects the immune-pathogenesis by inducing cytotoxicity, which results in thyrocyte death and thyroid atrophy (5). Therefore, it could be theorized that anti-TPO may increase the risk of hypothyroidism after treatment of GD. Unlike the restricted IgG type of TRAb, anti-TPO are polyclonal (6). It has been suggested that the presence of anti-TPO is secondary to the destruction of thyroid cells (6). Both patients with GD developing hypothyroidism and patients with autoimmune thyroiditis (AIT) progressing to GD have been observed (6). The occurrence of either disease form is common in family members of patients with GD (6).

There are three treatment options for GD. The favored treatment for uncomplicated GD in Europe is anti-thyroid drugs (ATD) (2), which aims at achieving a euthyroid state without the need of lifelong levothyroxine treatment (9). ATD inhibit thyroid peroxidase, thus blocking the synthesis of thyroxine (T4) and triiodothyronine (T3) (2). The main drawback of ATD is the high recurrence rate (10). Radioiodine ablation (RI) or thyroidectomy represent other treatment options (2) with lower relapse rates but with the drawback of achieving permanent hypothyroidism, requiring lifelong levothyroxine substitution (11). TRAb levels usually decrease during ATD treatment and are used to assess treatment success (9).

In a Swedish study from 2019, 58.9% of the patients with GD were cured after a single ATD treatment period of typically 12-18 months (11). However, 23%-26% of the patients developed autoimmune hypothyroidism after ATD cessation (3, 11). After ATD treatment, 34%-40.3% of the patients maintained a euthyroid state without requiring levothyroxine treatment after 6-25 years follow-up (3, 11).

The presence of anti-TPO at diagnosis of GD has been reported to increase remission rates in GD (12, 13). However, these studies had a short follow-up, and further studies are needed to confirm the correlation. The impact of the presence of anti-TPO at diagnosis of GD on the risk of developing hypothyroidism after ATD remains unknown, information that is important for individualized treatment.

The aims of this study were to 1) investigate whether elevated anti-TPO levels at diagnosis of GD lower the risk of relapse after ATD or RI treatment, and 2) determine whether elevated anti-TPO levels at diagnosis of GD increase the risk of developing hypothyroidism after ATD treatment.

## 2 Materials and methods

This was an observational, non-interventional, single-center retrospective registry study. Patients from the GD2002 study (patients with newly diagnosed GD at Malmö University Hospital 2003-2018) (14), were prospectively included in a database. Among other parameters, data on age, sex, place of birth, smoking status, presence of ophthalmopathy at diagnosis, and the levels of TRAb, anti-TPO, free T3, free T4, and TSH at diagnosis were registered (14, 15). For the current study, additional data on treatment modality, outcomes (including relapse in GD or hypothyroidism after ATD treatment), duration of ATD treatment, duration of follow-up, and concomitant autoimmune disorders were extracted from the patient records.

Thyrotoxicosis was defined by the presence of clinical symptoms of hyperthyroidism, plasma TSH concentrations <0.2 mIU/L, and elevated plasma levels of free T4 and/or free T3 (14). GD was confirmed through TRAb-positivity or a diffuse uptake on technetium scintigraphy (14).

Patients were excluded from the analyses if they met any of the following criteria: subclinical hyperthyroidism (defined as TSH <0.40 mIU/L with normal levels of free T3 and free T4), hypothyroidism before the diagnosis of GD, previous thyrotoxicosis, spontaneous remission of GD, surgery as first-line choice of treatment, not followed at Malmö University Hospital, and cases where the duration of ATD could not be determined. In addition, patients with relapse in GD and patients undergoing thyroidectomy or RI were excluded from the analysis of development of hypothyroidism after ATD.

To be categorized as treated with ATD, ATD had to be continued for at least 6 months to exclude patients discontinuing treatment early or those receiving ATD solely as a bridge to other treatments. To be categorized as treated with RI, the use of ATD for ≥6 months was not allowed. Relapse in GD (used synonymously with recurrence) was defined as suppressed TSH requiring treatment at any timepoint after end of ATD treatment. Temporarily suppressed TSH levels that normalized spontaneously were not classified as relapse. In this observational study, the choice of treatment modality was based on center practices, contemporary medical knowledge, patient’s reproductive intentions, and personal preferences.

Hypothyroidism was defined as elevated TSH and decreased T3 and/or T4 levels requiring levothyroxine substitution. Subclinical hypothyroidism was defined as an elevated TSH with normal peripheral hormones in the absence of symptoms or need for treatment and was not included as hypothyroidism in the analyses. Anti-TPO positivity at diagnosis of GD was defined as an antibody level of ≥35 kIU/L. The TRAb values were divided into quartiles (due to method changes resulting in different reference limits during the study period) for the analysis of relapse and were assessed as either above or below median levels for the analysis of hypothyroidism development. A *post-hoc* analysis was performed to assess any association between anti-TPO values at ± 6 months of ATD cessation and development of hypothyroidism.

**Figure 1:**
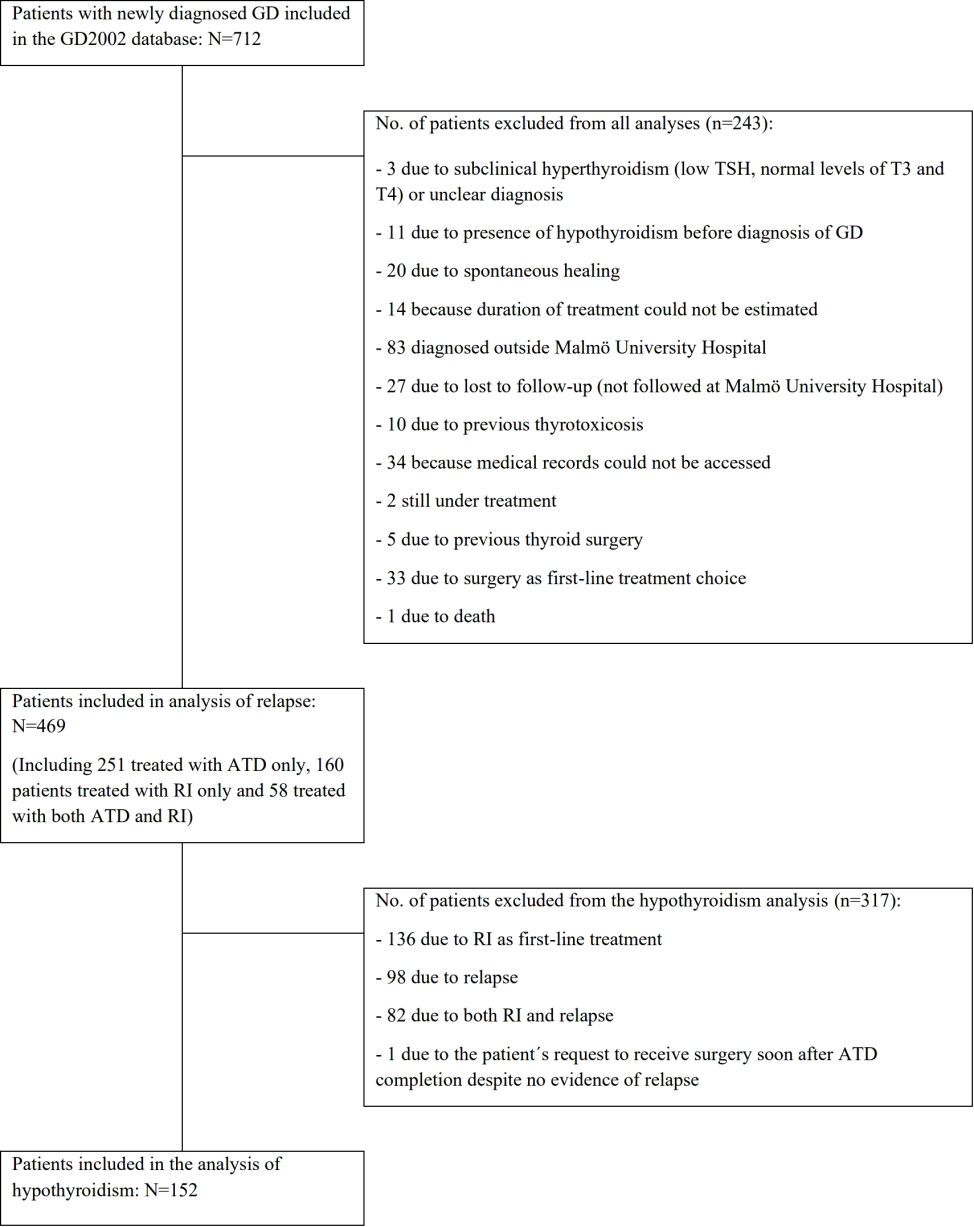
Patients included in the analyses and reasons for exclusions. ATD, antithyroid drugs; GD, Grave’s disease; RI, radioactive iodine ablation; T3, triiodothyronine; T4, thyroxine; TSH, thyroid-stimulating hormone.

712 unique patients were identified from the GD2002 database, of whom 469 were included in the analyses in this study. The analysis of development of hypothyroidism after ATD included 152 patients (**Figure 1**).

### 2.1 Statistics

The normality of the distribution was assessed using the Shapiro-Wilk test. The Pearson’s χ2 test was used to compare categorical variables and the Mann-Whitney U test for comparing continuous variables. A binary logistic regression model was used to determine the odds ratio (OR) of having a relapse of GD after ATD or RI treatment and the OR of developing hypothyroidism after ATD. Estimates are presented with their corresponding 95% confidence intervals (CIs). Statistical analyses were performed using SPSS version 23.0 (SPSS, IBM, Armonk, NY, USA) and GraphPad Prism version 8.0a (GraphPad Software, Inc., San Diego, CA, USA). A two-sided p-value of <0.05 was considered statistically significant.

### 2.2 Ethics

This study was performed according to the principles of the Declaration of Helsinki and was approved by The Regional Ethical committee in Lund, (GD2002) Dnr LU585/2006.

## 3 Results

At the time of diagnosis, approximately one-third of the 469 patients included in the analysis of relapse were below 40 years, and 80.8% were female. Half of the patients (48.4%) were never-smokers at diagnosis. Two thirds of the patients (65.9%) were anti-TPO positive at diagnosis of GD. Approximately one-fifth of the patients (18.3%) had ophthalmopathy at diagnosis. Concurrent autoimmune disease was present in 14.7% of patients (**Table 1**). For patient characteristics at diagnosis of the 152 patients included in the analysis of hypothyroidism, see **Table 2**.

**Table 1:** Patient characteristics at diagnosis of the 469 patients included in the analysis of relapse.

Variable		N=469
Age (years)	<40; n (%)	158 (33.7%)
	≥40; n (%)	311 (66.3%)
	Mean	47.4
	Median (Min-max)	48 (12^a^-90)
Sex	Male; n (%)	90 (19.2%)
	Female; n (%)	379 (80.8%)
Smoking	Never; n (%)	227 (48.4%)
	Previous; n (%)	94 (20.0%)
	Current; n (%)	138 (29.4%)
	Missing; n (%)	10 (2.1%)
Anti-TPO	Mean	479.0
	Median (Min-max)	142 (0-7010)
	Yes (≥35 kIU/L); n (%)	309 (65.9%)
	No (<35 kIU/L); n (%)	153 (32.6%)
	Missing; n (%)	7 (1.5%)
Autoimmune disease other than GD	Yes; n (%)	69 (14.7%)
	No; n (%)	400 (85.3%)
Ophthalmopathy	Yes; n (%)	86 (18.3%)
	No; n (%)	381 (81.2%)
	Missing; n (%)	2 (0.4%)

a Four patients were below 18 years at diagnosis, all of whom were diagnosed at the Pediatric Department at Malmö University Hospital and have been followed for several years at the Endocrinology department at Malmö University Hospital from 18 years of age.

Anti-TPO, anti-thyroid peroxidase antibodies; GD, Grave’s disease; N, number of patients in the analysis set; n, number of patients within the category.

**Table 2:** Patient characteristics at diagnosis of the 152 patients included in the analysis of hypothyroidism.

Variable		N=152
Age (years)	<40; n (%)	50 (32.9%)
	≥40; n (%)	102 (67.1%)
	Mean	45.8
	Median (Min-max)	48 (14 a -80)
Sex	Male; n (%)	30 (19.7%)
	Female; n (%)	122 (80.3%)
Smoking	Never; n (%)	77 (50.7%)
	Previous; n (%)	34 (22.4%)
	Current; n (%)	38 (25.0%)
	Missing; n (%)	3 (2.0%)
Anti-TPO	Mean	376.7
	Median (Min-max)	134 (0-2990)
	Yes (≥35 kIU/L); n (%)	98 (64.5%)
	No (<35 kIU/L); n (%)	53 (34.9%)
	Missing; n (%)	1 (0.7%)
Autoimmune disease other than GD	Yes; n (%)	15 (9.9%)
	No; n (%)	137 (90.1%)
Ophthalmopathy	Yes; n (%)	32 (21.1%)
	No; n (%)	119 (78.3%)
	Missing; n (%)	1 (0.7%)

a Three patients were below 18 years at diagnosis, all of whom were diagnosed at the Pediatric Department at Malmö University Hospital and have been followed for several years at the Endocrinology department at Malmö University Hospital from 18 years of age.

Anti-TPO, anti-thyroid peroxidase antibodies; GD, Grave’s disease; N, number of patients in the analysis set; n, number of patients within the category.

**Figure 2:**
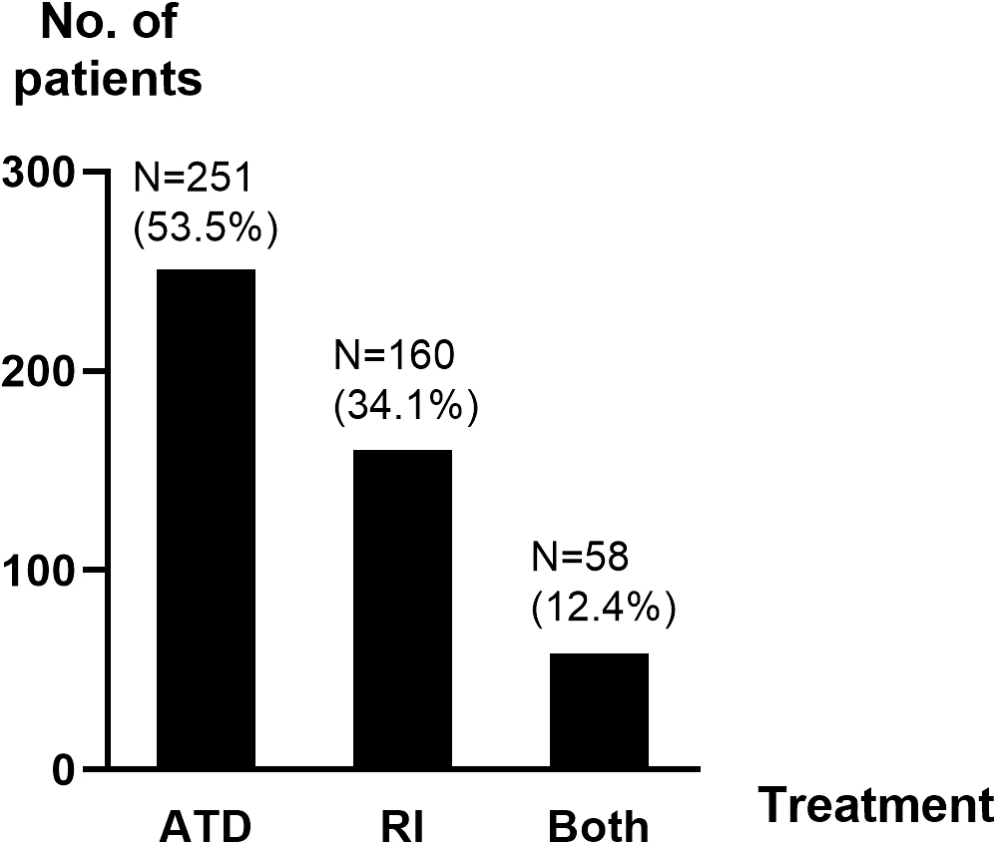
Distribution of main treatment. ATD, antithyroid drugs; N, number of patients; RI, radioactive iodine ablation.

Over half of the patients (53.5%) received ATD for at least 6 months after the diagnosis of GD and 34.1% were primarily treated with RI (**Figure 2**). The mean duration of follow-up was 5.9 years (range: 0 months-18.4 years) from end of treatment for patients receiving ATD and 5.2 years (range: 3 months-19.6 years) from diagnosis for those receiving RI.

Among patients treated with ATD, relapse of GD occurred in 95 (37.8%) individuals. Of these, 38 relapsed within the first 6 months after treatment cessation, and 57 relapsed after 7 or more months (mean: 26.8 months; median: 12 months; range: 0-205 months). The GD relapse rate after ATD was almost identical in patients with anti-TPO (37.0%) and those without anti-TPO (38.4%) at diagnosis of GD (p=0.839). Relapse after ATD was more common in patients younger than 40 years at diagnosis (52.7%) than in those aged 40 years and above (26.2%), (p<0.0001).

No statistically significant differences were seen in relapse rate with respect to sex, concurrent autoimmune diseases, TRAb-levels at diagnosis, ophthalmopathy at diagnosis, or duration of ATD treatment. Patients with relapse had a longer follow-up than those without (**Table 3**). The univariate analysis confirmed that younger age at diagnosis was associated with a higher likelihood of relapse (OR: 0.96; 95% CI: 0.95-0.99, p<0.01). Furthermore, current smokers were significantly more likely to relapse than former smokers (OR: 2.63; 95% CI: 1.18-5.88, p<0.05). Age and smoking status also demonstrated statistically significant differences in the multivariate model.

**Table 3:** GD relapse rate in patients treated with ATD.

		Total	Relapse	No relapse	p-value
			n (%)	n (%)	
Any		N=251	95 (37.8%)	156 (62.2%)	
Age at diagnosis of GD (years)	<40	N=110	58 (52.7%)	52 (47.3%)	<0.0001^a^
	≥40	N=141	37 (26.2%)	104 (73.8%)	
Sex	Male	N=44	14 (31.8%)	30 (68.2%)	0.364^a^
	Female	N=207	81 (39.1%)	126 (60.9%)	
Smoking at diagnosis of GD	Never	N=125	47 (37.6%)	78 (62.4%)	0.057^a^
	Previous	N=48	12 (25.0%)	36 (75.0%)	
	Current	N=73	34 (46.6%)	39 (53.4%)	
TRAb at diagnosis of GD (IE/L)	>75^th^ percentile	N=37	14 (37.8%)	23 (62.2%)	0.776^a^
	50-75^th^ percentile	N=37	15 (40.5%)	22 (59.5%)	
	25-50^th^ percentile	N=42	17 (40.5%)	25 (59.5%)	
	<25^th^ percentile	N=48	15 (31.3%)	33 (68.8%)	
Anti-TPO at diagnosis of GD	Yes, ≥35 kIE/L	N=162	60 (37.0%)	102 (63.0%)	0.839^a^
	No, <35 kIE/L	N= 86	33 (38.4%)	53 (61.6%)	
Ophthalmopathy at diagnosis of GD	Yes	N=56	22 (39.3%)	34 (60.7%)	0.822^a^
	No	N=194	73 (37.6%)	121 (62.4%)	
Autoimmune disease other than GD at diagnosis of GD	Yes	N=30	13 (43.3%)	17 (56.7%)	0.509^a^
	No	N=221	82 (37.1%)	139 (62.9%)	
Duration of ATD (months)	6-18	N=112	39 (34.8%)	73 (65.2%)	0.166^a^
	19-24	N=86	30 (34.9%)	56 (65.1%)	
	>24	N=53	26 (49.1%)	27 (50.9%)	
Duration of follow- up from end of treatment (months)	Median (Min-Max)		104 (3-221)	41.5 (0-220)	<0.001^b^

a Chi-square test;

b Mann-Whitney test.

Anti-TPO, anti-thyroid peroxidase antibodies; ATD, antithyroid drugs; GD, Grave’s disease; N, number of patients in the analysis set; n, number of patients within the category; TRAb, thyroid-stimulating hormone receptor antibodies.

**Figure 3:**
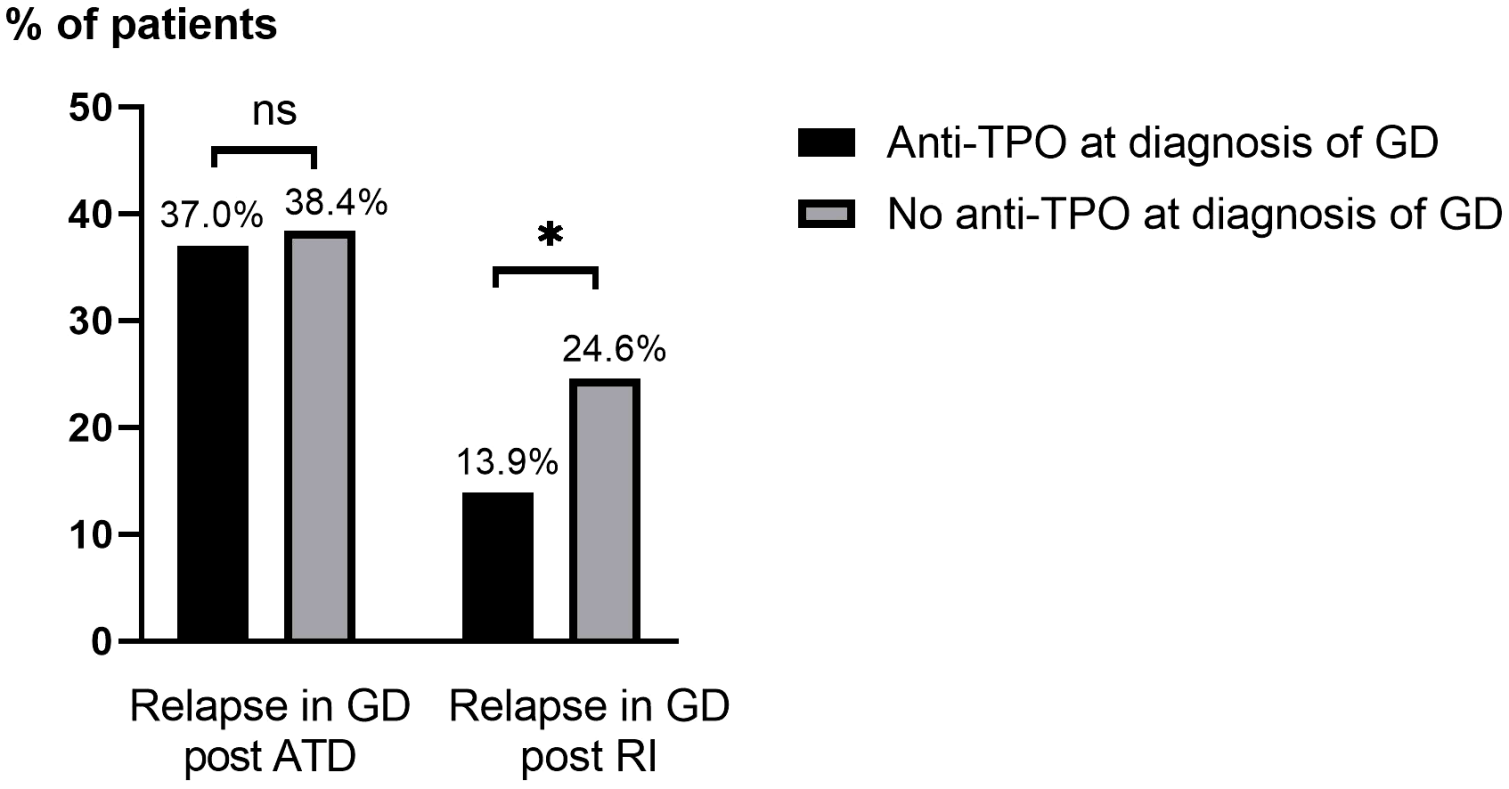
Relapse of GD after ATD and RI treatment based on the presence of anti-TPO at diagnosis of GD. Anti-TPO, anti-thyroid peroxidase; ATD, antithyroid drugs; GD, Grave’s disease; ns, not statistically significant (p>0.05); RI, radioactive iodine ablation. *p=<0.05.

The relapse rate of GD in patients treated with RI (total 18.1%) was lower in patients with anti-TPO at diagnosis of GD (13.9%) than in those without (24.6%), p=0.049, **Figure 3**. There was a trend towards patients aged ≥40 years being overrepresented among those with relapse after receiving RI (p=0.088). No statistically significant differences were seen in relapse rate in relation to sex, concurrent autoimmune diseases, smoking habits, ophthalmopathy at diagnosis, or duration of follow-up after RI (**Table 4**). In patients treated with RI, the results obtained with the univariate model showed that relapse was less common in patients with anti-TPO at diagnosis than in those without (OR: 0.41; 95% CI: 0.17-0.96, p<0.05) which is consistent with the results obtained with the Chi-square test.

**Table 4:** GD relapse rate in patients treated with RI.

		Total	Relapse	No relapse	p-value
			n (%)	n (%)	
Any		N=160	29 (18.1%)	131 (81.9)	
Age at diagnosis of GD (years)	<40	N=21	1 (4.8%)	20 (95.2%)	0.088^a^
	≥40	N=139	28 (20.1%)	111 (79.9%)	
Sex	Male	N=34	5 (14.7%)	29 (85.3%)	0.560^a^
	Female	N=126	24 (19.0%)	102 (81.0%)	
Smoking at diagnosis	Never	N=73	13(17.8%)	60 (82.2%)	0.794^a^
	Previous	N=36	8 (22.2%)	28 (77.8%)	
	Current	N=48	8 (16.7%)	40 (83.3%)	
TRAb at diagnosis of GD (IE/L)	>75^th^ percentile	N=37	4 (10.8%)	33 (89.2%)	0.139^a^
	50-75^th^ percentile	N=35	4 (11.4%)	31 (88.6%)	
	25-50^th^ percentile	N=41	10 (24.4%)	31 (75.6%)	
	<25^th^ percentile	N=32	9 (28.1%)	23 (71.9%)	
Anti-TPO at diagnosis of GD	Yes, ≥35 kIE/L	N=101	14 (13.9%)	87 (86.1%)	0.049^a^
	No, <35kIE/L	N= 57	14 (24.6%)	43 (75.4%)	
Ophthalmopathy at diagnosis of GD	Yes	N=26	5 (19.2%)	21 (80.8%)	0.886^a^
	No	N=133	24 (18.0%)	109 (82.0%)	
Autoimmune disease other than GD at diagnosis of GD	Yes	N=29	3 (10.3%)	26 (89.7%)	0.229^a^
	No	N=131	26 (19.8%)	105 (80.2%)	
Duration of follow-up from diagnosis (months)	Median (Min-Max)		57 (4-235)	49 (3-177)	0.257^b^

a Chi-square test;

b Mann-Whitney test.

RI, radioactive iodine ablation; Anti-TPO, anti-thyroid peroxidase antibodies; GD, Grave’s disease; N, number of patients in the analysis set; n, number of patients within the category; TRAb, thyroid-stimulating hormone receptor antibodies.

**Figure 4:**
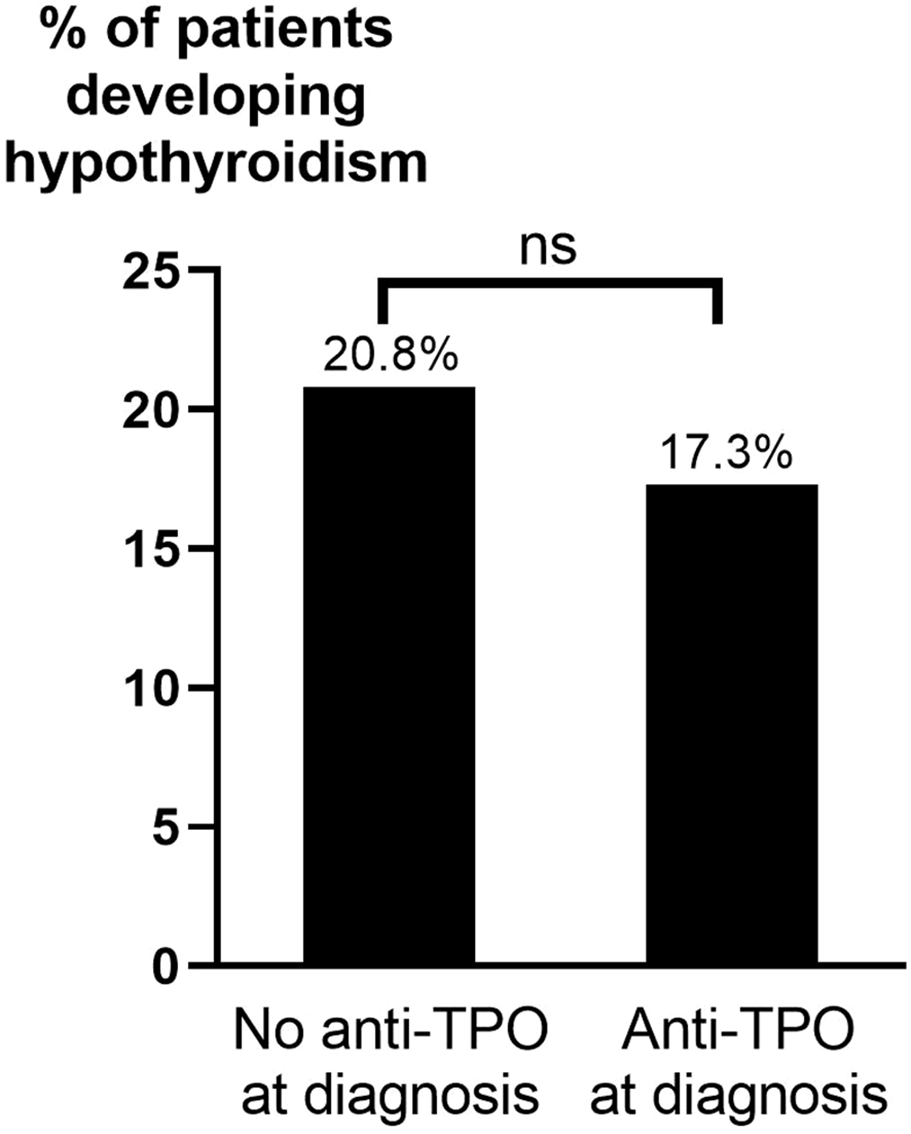
Hypothyroidism after ATD by presence of anti-TPO at diagnosis of GD. ATD, anti-thyroid drugs; Anti-TPO, anti-thyroid peroxidase antibodies; ns, not statistically significant (p>0.05).

Patients receiving ATD as single treatment for >24 months were overrepresented among those who developed hypothyroidism. Hypothyroidism after ATD occurred in 17.3% of patients with anti-TPO at diagnosis of GD compared to 20.8% without (**Figure 4**). This difference was not statistically significant (p=0.471). No statistically significant differences in development of hypothyroidism after ATD were seen based on age, sex, smoking habits, TRAb, anti-TPO levels, or ophthalmopathy at diagnosis (**Table 5**). The results of an univariate analysis were consistent with the results obtained with the Chi-squared test and showed that hypothyroidism after ATD was more common among patients treated for >24 months than those treated for 6-18 months (OR: 4.55; 95% CI: 1.64-14.29, p<0.05) or 19-24 months (OR: 3.57; 95% CI: 1.23-10.0, p<0.01). Additionally, 79 patients had anti-TPO measured at ± 6 months of ATD cessation. Among those, 14 patients (17.7%) developed hypothyroidism, 7 of 38 (18.4%) who were anti-TPO positive and 7 of 41 (17.1%) who were anti-TPO negative (p=0.875).

**Table 5:** Development of hypothyroidism after cessation of ATD treatment of GD.

		Total	Hypothyroidism	No hypothyroidism	p-value
			n/N (%)	n/N (%)	
Any		N=152	29 (19.1%)	123 (80.9%)	
Age at diagnosis of GD (years)	<40	N=50	11 (22.0%)	39 (78.0%)	0.521^a^
	≥40	N=102	18 (17.6%)	84 (82.4%)	
Sex	Male	N=30	3 (10.0%)	27 (90.0%)	0.158^a^
	Female	N=122	26 (21.3%)	96 (78.7%)	
Smoking at diagnosis of GD	Never	N=77	17 (22.1%)	60 (77.9%)	0.323^a^
	Previous	N=34	3 (8.8%)	31 (91.2%)	
	Current	N=38	9 (23.7%)	29 (76.3%)	
TRAb at diagnosis of GD	Above median	N=50	11 (22.0%)	39 (78.0%)	0.298^a^
	Below median	N=50	7 (14.0%)	43 (86.0%)	
Anti-TPO at diagnosis of GD	Yes, ≥35 kIE/L	N=98	17 (17.3%)	81 (82.7%)	0.471^a^
	No, <35k IE/L	N=53	11 (20.8%)	42 (79.2%)	
Ophthalmopathy at diagnosis of GD	Yes	N=32	9 (28.1%)	23 (71.9%)	0.149^a^
	No	N=119	20 (16.8%)	99 (83.2%)	
Autoimmune disease other than GD at diagnosis of GD	Yes	N=15	4 (26.7%)	11 (73.3%)	0.431^a^
	No	N=137	25 (18.2%)	112 (81.8%)	
Duration of ATD (months)	6-18	N=70	9 (12.9%)	61 (87.1%)	0.006^a^
	19-24	N=55	9 (16.4%)	46 (83.6%)	
	>24	N=27	11 (40.7%)	16 (59.3%)	
Duration of follow-up from end of treatment (months)	Median (Min-Max)		57 (0-220)	36 (2-183)	0.030^b^

a Chi-square test;

b Mann-Whitney test.

Anti-TPO, anti-thyroid peroxidase antibodies; ATD, antithyroid drugs; GD, Grave’s disease; N, number of patients in the analysis set; n, number of patients within the category; TRAb, thyroid-stimulating hormone receptor antibodies.

## 4 Discussion

We hypothesized that anti-TPO at diagnosis of GD would decrease the risk of relapse following treatment while increasing the risk of hypothyroidism post-treatment. However, no difference in relapse rate after treatment with ATD was observed between patients with or without anti-TPO at diagnosis of GD. Interestingly, the presence of anti-TPO at diagnosis of GD reduced the risk of relapse after RI treatment. Previous published studies focused solely on ATD treatment outcomes (12, 13). Schott et al. reported that patients with decreasing titers of TRAb and anti-TPO during treatment had an increased chance of remission (13). Notably, their study documented a relapse rate nearly twice as high as in our study. Furthermore, initial blood samples in that study were taken on average four months after diagnosis (13), making it difficult to compare with our results.

Muir et al. found that the absence of anti-TPO increased the risk of GD relapse after ATD (OR: 2.21) (12). They hypothesized that since anti-TPO cause hypothyroidism by thyrocyte damage from lymphocytic infiltration, this process would protect against relapse of GD after the cessation of ATD treatment (12). However, according to that theory, patients with anti-TPO should also be more likely to develop hypothyroidism.

Our overall relapse rate of 37.8% after ATD (mean follow-up of 5.9 years from cessation of ATD treatment) is at the lower end of previously reported relapse rates of 30%-70% (11, 12, 16, 17). Since the patients were not randomized to a certain treatment, the patients with a low chance of remission probably have, after assessment of individual clinical parameters, received RI, surgery, or long-term ATD treatment.

Relapse occurred in 34.8% of patients who had ATD for 6-18 months vs 49.1% of patients who had ATD for >24 months (p=0.166) This could reflect the physician´s hesitation to stop treatment after 18-24 months due to persistently elevated TRAb levels or other factors indicating disease activity. Interestingly, while a Cochrane review from 2010 did not find any benefit from prolonged treatment (18), another systematic review and meta-analysis found that prolonged use of ATD increased remission rates (10). Based on a recent study, Azizi et al. conclude that long-term ATD treatment (>60 months of continuous treatment) is both safe and effective. They recommend long-term ATD for most patients with GD to avoid relapse and prefer this treatment over RI after relapse (19). Our finding that ATD treatment for >24 months emerged as a risk factor for the development of hypothyroidism suggests that prolonged treatment needs to be further evaluated. If longer ATD therapy reduces the risk of relapse but at the same time increases the risk of hypothyroidism, the advantages of ATD compared to RI and surgery are reduced.

With the intention of the RI treatment being ablation, recurrence after RI can be considered a treatment failure. The proportion of patients not cured in the current registry study (18.1%) as well as in published literature (18.5%-21.5%) (11, 20) is high. Therefore, the patient should be informed that, although being considerably lower than after ATD, there is a risk of not achieving remission after a single RI treatment.

Age proved to be a significant factor in treatment outcomes. Relapse after treatment with ATD was significantly more common among patients aged <40 years at diagnosis than patients aged ≥40 years, a finding corroborated by others (16, 21). The opposite trend was observed after RI, however, the number of patients under 40 years of age was low since RI is more commonly used in older patients.

In the current study, 14.7% of the patients had a concurrent autoimmune disease, which is similar to published data (16.7%) (4). However, no correlation was observed between concurrent autoimmune disease and treatment outcomes.

We defined relapse as suppressed TSH requiring treatment at any timepoint after end of treatment. This definition has also been used by Muir et al. (12), while other studies only considered it as relapse if occurring more than 6 months (22) or 12 months (9) after the cessation of ATD treatment. By including relapse occurring at any timepoint after end of treatment, we achieved information about the efficacy of the treatment, however, we might have defined patients who never became disease-free as relapsed. This approach could have overestimated the relapse rate compared to other studies using different definitions but may better illustrate the patient’s chance of cure. Our study followed patients for up to 19.6 years, however, the mean follow-up time was 5.9 years after ATD since patients were referred to primary care once thyroid values were stabilized (euthyroid state or treated hypothyroidism). With a few exceptions (3, 11), studies with longer follow-up than 2-5 years are scarce (3).

A limitation of this study is the lack of information on goiter size, a well-known risk factor for relapse in GD. Furthermore, the number of patients included in the analysis of hypothyroidism after ATD was low despite starting with a large cohort, making it difficult to prove any statistically significant differences between groups.

Contrary to our hypothesis, anti-TPO at diagnosis did not correlate with an increased rate of hypothyroidism after ATD cessation. This may indicate that anti-TPO could be an incidental marker of transient thyroid damage (6) rather than a direct contributor to hypothyroidism development. A *post-hoc* analysis (N=79) showed no association between TPO positivity at ±6 months of ATD cessation and development of hypothyroidism. Future studies with a larger sample size should explore whether elevated anti-TPO levels at the end of treatment, particularly in combination with normalized TRAb values, increase the risk of hypothyroidism.

Knowing whether levels of anti-TPO, TRAb and other factors affect relapse rate or hypothyroidism after treatment for GD can help the physician make informed decisions on choice of treatment. Our study supports earlier findings with young age being a risk factor for relapse after ATD. Smoking is another known risk factor, and we found borderline significance for current smoking being more common in patients with relapse after ATD. Based on the findings of the current study, it is not necessary for clinicians to consider anti-TPO levels at GD diagnosis as a risk factor for relapse or hypothyroidism after ATD. This is consistent with this variable not being included in the GREAT score (21). The GREAT score, which predicts the risk of relapse of GD after ATD therapy by combining clinical markers such as age, serum concentration of free T4, serum thyrotropin-binding inhibitory immunoglobulin (TBII), and goiter size (21), remains to date the most accurate predictive model of recurrence after ATD.

## 5 Conclusion

In conclusion, our study showed that the presence of anti-TPO antibodies at GD diagnosis did not influence the relapse rate after ATD treatment, nor did it correlate with the development of hypothyroidism post-ATD. Conversely, for patients receiving RI treatment, the presence of anti-TPO at diagnosis was associated with a reduced risk of relapse. Duration of ATD treatment >24 months emerged as a risk factor for the development of hypothyroidism. Increased knowledge about predictive factors is crucial for tailoring personalized treatment strategies and optimizing patient outcomes.

## Data Availability

The datasets presented in this article are not readily available because the ethical permit for this study and local laws (GDPR) do not allow distribution of non-aggregated patient information. Specific questions can be discussed upon request. Requests to access the datasets should be directed to tereza.planck@med.lu.se.
